# The oxidative-stress-senescence axis in keratoconus: new insights into corneal degeneration

**DOI:** 10.3389/fmolb.2025.1539542

**Published:** 2025-04-24

**Authors:** Maria Laura Passaro, Michele Rinaldi, Valentina Morgera, Antonia Feola, Vito Romano, Mario Troisi, Diego Strianese, Raffaele Piscopo, Samantha Messina, Antonella Romano, Antonio Porcellini, Antonio Pezone, Ciro Costagliola

**Affiliations:** ^1^ Department of Neurosciences, Reproductive Sciences and Dentistry, University of Naples “Federico II”, Naples, Italy; ^2^ Department of Medicine and Health Sciences “V. Tiberio”, University of Molise, Campobasso, Italy; ^3^ Department of Biology, University of Naples “Federico II”, Naples, Italy; ^4^ Department of Medical and Surgical Specialties, Radiological Sciences, and Public Health, Eye Clinic, ASST Spedali Civili di Brescia, University of Brescia, Brescia, Italy; ^5^ Department of Science, Roma Tre University, Rome, Italy

**Keywords:** keratoconus, oxidative stress, mitochondrial dysfunction, senescence, inflammation, antioxidant therapy

## Abstract

Keratoconus is a bilateral and asymmetric degenerative eye disease that causes corneal thinning and bowing, leading to irregular astigmatism and vision loss. Although environmental and genetic factors contribute to the disease’s development, the exact cause and underlying pathological mechanism remain unknown. In this review, we comprehensively explore the latest pathophysiological mechanisms of keratoconus, focusing on oxidative damage and inflammation. Senescence emerges as a key driver of keratoconus pathogenesis. Understanding these common elements enhances our understanding of the disease and paves the way for innovative therapeutic approaches to keratoconus.

## 1 Introduction

Keratoconus (KC) is a bilateral and asymmetric ocular condition characterized by gradual corneal thinning and conical protrusion, resulting in irregular astigmatism and reduced visual acuity (Santodomingo-Rubido et al., 2022). Onset typically coincides with puberty, occurring in the late teens for males and early twenties for females, with progression continuing until the fourth decade of life when stability is usually reached (Santodomingo-Rubido et al., 2022; Arora and Lohchab, 2019). While once deemed uncommon, the incidence of KC has witnessed an upward trend in recent decades. Prevalence estimates indicate a notable increase, with figures suggesting a prevalence of approximately 1.7% in the United States, ranging from 0.9% to 2.3% in developing nations such as China (Hu et al., 2023). A recent meta-analysis involving a cohort of 50 million individuals across 15 nations reported a global prevalence rate of 138 per 100,000 individuals (Hashemi et al., 2020). The last therapeutic option for advanced keratoconus is corneal transplantation, due to the extreme ectasia, thinning, and scarring, all of which seriously impede vision and represent a serious risk for corneal perforation. While the precise role that genetic and environmental factors play in the development of keratoconus are largely unclear and probably varied, they interact in a complicated way. Despite numerous studies, the cause of keratoconus remains poorly known.

Due to its function, the cornea is continuously exposed to light, including ultraviolet (UV) radiation. UV exposure generates ROS and RNS (Preiser, 2012), making it particularly vulnerable to oxidative stress. Even though KC was previously thought to be non-inflammatory, new research suggests that inflammatory elements may play a key role in the disease’s etiology. Patients with keratoconus have been found to have abnormal levels of antioxidant enzymes (Wojcik et al., 2013), increased levels of mitochondrial DNA damage (Chalimeswamy et al., 2022; Vallabh et al., 2017a; Cejková and Cejka, 2015), accumulation of cytotoxic byproducts from the lipid peroxidation and nitric oxide (NO) pathways, and increased levels of pro-inflammatory cytokines in their tears and corneas (Zhang et al., 2021).

Moreover, studies conducted *in vitro* have discovered that cultivated keratoconus corneal fibroblasts produce more reactive nitrogen species (RNS) and reactive oxygen species (ROS) at basal levels. Furthermore, compared to normal fibroblasts, they were more vulnerable to oxidative stresses. Elevated oxidative stress has been implicated in physiological conditions such as aging and exercise, and in various pathological conditions, including cancer, neurodegenerative diseases, cardiovascular diseases, diabetes, inflammatory diseases, and intoxications (Preiser, 2012; Hajam et al., 2022). However, in the context of ocular diseases, increasing evidence supports the role of oxidative stress (Zhang et al., 2024; Wen et al., 2024; Van Eijgen et al., 2024; Passaro et al., 2023; Sun et al., 2024), without clarifying their sources and consequences.

This review will thoroughly analyze all aspects of this complex disease, whose etiology is yet unknown.

## 2 Corneal structure

**FIGURE 1:**
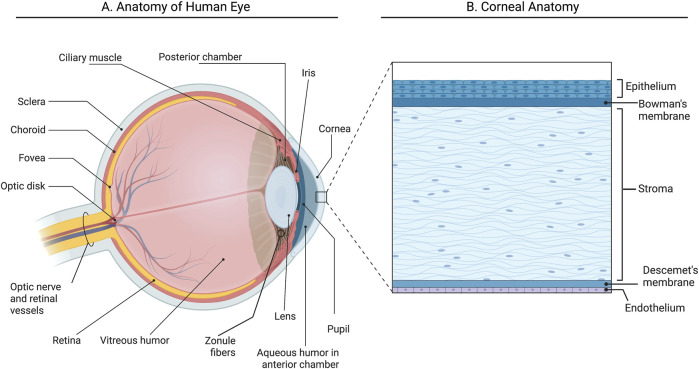
Anatomy of the human eye and cornea. **(A)** Section of the anterior part of the human eye; **(B)** Section of the cornea illustrating five layers. Created with Biorender.com.

The human cornea plays a dual role as a protective barrier for the eye and a key refractive surface essential for vision (Eghrari et al., 2015). The cornea is a dome-shaped transparent structure, and its shape and clarity, are the main characteristics enabling such great refractive power. The cornea, being avascular, obtains its nutrients from the tear film, the aqueous humour and blood vessels at the peripheral edge of the cornea (Figure 1A). Human corneal transparency is the result of several related factors: avascularity, structural regularity of the covering epithelium, regular arrangement of the extracellular and cellular components in the stroma and functionality of the endothelium to regulate corneal hydration (Nita and Grzybowski, 2016). It comprises five distinct layers: from anterior to posterior; these include the epithelium, Bowman’s layer, the collagen-rich stroma, Descemet’s membrane, and the endothelial layer (Volatier et al., 2020) (Figure 1B). Measuring approximately 500 μm and representing about 90% of the corneal axis, the stroma is a hydrated extracellular matrix composed of type I and V collagen, interwoven with glycosaminoglycans that regulate hydration and structural integrity (Torricelli and Wilson, 2014). Collagen fibrils are highly organized into lamellae, contributing to the cornea’s shape and transparency by allowing light to pass through the collagen fibril framework without scattering (Boote et al., 2011). The extracellular matrix (ECM) within the stroma is maintained by specialized fibroblasts known as keratocytes, which remain dormant until activated by growth factors (e.g., TGFβ, FGF, PDGF) in response to injury (Ljubimov and Saghizadeh, 2015). Activated keratocytes differentiate into fibroblasts and myofibroblasts, aiding wound healing by producing ECM-degrading enzymes and contracting to close wounds. These processes can result in corneal haze and scarring, primarily due to the loss of water-soluble proteins that decrease light scattering, including aldehyde dehydrogenase class 3, transketolase, and alpha-enolase (Ljubimov and Saghizadeh, 2015; Andrew Cuthbertson et al., 1992; Jester et al., 1999). The corneal epithelium, posteriorly supported by the basement membrane and Bowman’s layer, acts as the main barrier to fluids and pathogens and secretes cytokines that influence keratocyte behaviour during wound healing (Eghrari et al., 2015; Volatier et al., 2020). Finally, the endothelium maintains stromal transparency by actively transporting water out of the stroma, through tight junctions and endothelial pumps, preventing excessive swelling (Figure 1) (Tuft and Coster, 1990).

Even small malfunctions in these components and/or impaired communication can compromise their function. The cornea’s particular physiology makes it vulnerable to oxidative damage, and various corneal diseases are influenced by oxidative damage, acquired and hereditary mitochondrial dysfunction, and other factors.

## 3 Keratoconus

Keratoconus is a corneal ectasic disorder characterized by protrusion and alteration of the central and paracentral cornea resulting in a conical shape, and subsequent progressive vision loss (Vohra et al., 2023). Common symptoms encompass reduced visual acuity, sensitivity to light, and visual distortions, among other manifestations. Key clinical features of keratoconus include irregular astigmatism, abnormal bulging of the cornea’s posterior surface, irregular thickness distribution across the cornea, and a non-inflammatory thinning of the corneal tissue (Gomes et al., 2015). Key clinical features suggesting keratoconus include challenges in correcting vision due to progressive myopia and astigmatism. Advanced cases can lead to unique signs like a v-shaped lower eyelid indentation during downward gaze, known as Münson sign, vertical lines located in the superficial stromal layers, known as Vogt’s striae, and an accumulation of ferritin in the epithelial layer encircling the bottom of the corneal protrusion, known as Fleischer’s ring (Volatier et al., 2020). Advanced stages may result in corneal hydrops, causing sudden vision loss. Furthermore, corneal scarring may occur due to spontaneous breaks in the cornea’s anterior limiting lamina (Asimellis and Kaufman, 2024). Diagnosing keratoconus involves meticulous refraction measurements and utilization of diagnostic tools like slit-lamp biomicroscope, corneal topography, tomography, and pachymetry. These techniques, particularly corneal tomography, are sensitive in detecting early signs of keratoconus and in tracking corneal shape changes in both the anterior and posterior curvature (Asimellis and Kaufman, 2024). Additional assessments include retinoscopy, keratometry, and examination of corneal epithelial thickness distribution using OCT devices (Asimellis and Kaufman, 2024).

Initially, the condition manifests unilaterally, but often, bilateral involvement occurs (Vohra et al., 2023). Although a definitive genetic link to keratoconus remains elusive, associations have been observed with systemic conditions such as Down syndrome, Leber congenital amaurosis, atopy, and connective tissue disorders like Ehlers-Danlos and Marfan syndromes (Vohra et al., 2023). Recent research by Chen et al. suggests an increased risk of keratoconus associated with hay fever, allergic rhinitis, eczema, and ulcerative colitis (Chen and Chen, 2024). Additionally, various genetic abnormalities have been identified in studies, implicating genes such as VSX1, TGFBI, LOX, COL5A1, and SOD1 (Bui et al., 2023). Factors such as excessive eye rubbing, atopy, and the use of hard contact lenses have also been implicated in exacerbating the condition.

**FIGURE 2:**
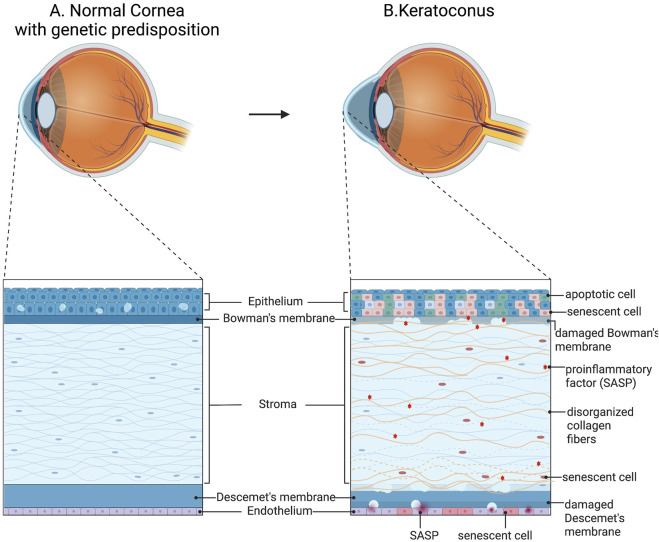
Schematic representation of cellular abnormalities in keratoconus. **(A)** Normal cornea structure con genetic predisposition. **(B)** Mechanical stretch in keratoconus enhances the expression of many protease genes in stromal cells, exacerbating ECM degradation. Additionally, aberrant differentiation of corneal epithelial cells and enhanced inflammatory signals were found. Created with BioRender.com.

In keratoconus, the aberrant behavior of keratocytes serves as a fundamental aspect of the pathology. Keratocytes exhibit heightened levels of endoplasmic reticulum, heightened apoptosis, and migration into the Bowman’s membrane (Polack, 1976). Consequently, the stroma undergoes damage and becomes more susceptible to external stresses. The survival of a limited number of keratocytes disrupts the ECM homeostasis, resulting in an altered composition with potentially senescent characteristics (Ku et al., 2008). The corneal stroma in KC is typified by decreased collagen lamellae, reduced amounts of microfibrillar material, and altered fibril arrangement, collectively leading to diminished mechanical resistance (Mocan et al., 2008; Rong et al., 2017) (Figure 2).

Furthermore, epithelial degeneration in keratoconus is evident, characterized by blebbing, reduced cell density, and thinning (Uçakhan et al., 2006; Jongebloed and Worst, 1987). In advanced stages, breakdown of the cell membrane results in the loss of basal epithelial cells, leaving behind flattened superficial epithelial cells resting on an altered basement membrane. Concurrently, degeneration of basal epithelial cells may trigger the release of proteolytic enzymes, exacerbating keratocyte damage and stromal cell vulnerability. Corneal thinning in keratoconus may stem from increased levels of degradative enzymes such as acid esterases, acid phosphatases, and acid lipases, coupled with elevated cathepsins B and G and reduced levels of protease inhibitors like alpha-1 protease inhibitors and alpha-2 macroglobulin (Joseph et al., 2011). This imbalance leads to excessive protease activity, damaging corneal tissue and contributing to thinning. Additionally, abnormal activity of corneal collagenase and an imbalance between matrix metalloproteinases (MMPs) and tissue inhibitors of MMPs (TIMPs) may further contribute to corneal thinning, leading to the destruction of ECM (Kenney et al., 2005). In this regard, matrix degradation and altered or abnormal levels of fibronectin and type VI collagen in KC corneas have been demonstrated as consequences (Kenney et al., 2005). Ruptures in the Bowman’s layer further compromise stromal integrity, while disruptions in Descemet’s membrane can impact the posterior stroma, resulting in fluid infiltration, altering cellular environments, influencing keratocyte behaviour, and ultimately leading to hydrops, subsequent scarring, and impaired visual quality (Sykakis et al., 2012; Fan et al., 2014) (Figure 2).

## 4 Is keratoconus really a non-inflammatory disease?

Keratoconus has been classically defined as a progressive, non-inflammatory, corneal ectatic condition (Santodomingo-Rubido et al., 2022). However, evidence from recent years suggests chronic inflammation may contribute to the progression of KC (Erdinest et al., 2023). As early as 2012, studies indicated a decrease in total protein levels in the tear film of KC patients compared to normal subjects (Balasubramanian et al., 2012a). Research by Balasubramanian et al. showed a significant reduction in secretory immunoglobulin A and dysregulation of lactoferrin, unrelated to contact lens wear (Balasubramanian et al., 2012a). Furthermore, Acera et al. found higher serum albumin levels in KC patients than in control subjects, and presence of serum albumin in KC tears, indicating blood-ocular barrier failure and suggesting conjunctival inflammation (Acera et al., 2011). Moreover, the increased expression of MMP-1, MMP-3, MMP-7, MMP-9, MMP-13, IL-4, IL-5, IL-6, IL-8, IL-17, TNF-α, and TNF-β in the tears of KC patients indicates that inflammatory and subsequent tissue degenerative processes play a significant role in the thinning and weakening of corneal connective tissue, contributing to the progressive degeneration observed in KC corneal structure (Balasubramanian et al., 2012b; Jun et al., 2011; Marques et al., 2023; Nichani et al., 2023; Peyman et al., 2021; Taurone et al., 2021). In 2021, Zhang et al. supported the hypothesis that inflammation underlies KC by conducting a meta-analysis, which revealed increased levels of proinflammatory cytokines IL-1β, IL-6, and TNF-α. This finding indicates significant changes in the cytokine profile in the tears of KC patients (Zhang et al., 2021). Later, a recent metanalysis revealed that KC patients showed elevated levels of inflammatory factors (e.g., IL1A, IL1B, IL6, TNF), a collagen-degrading enzyme (MMP9), and an apoptosis-related protein (SFRP1), while the expression levels of extracellular matrix -related proteins (e.g., LOX, MRC2, FMOD, and KERA) were reduced. This suggests abnormalities in KC inflammatory responses, matrix metabolism, and apoptotic processes (Song et al., 2024). Additionally, several studies have presented immunohistochemical evidence of inflammation in KC corneas, demonstrating cellular infiltration by macrophages, leukocyte accumulation, and dendritic Langerhans cells (Fan et al., 2015; Mandathara et al., 2018). In 2020, Loh et al. utilized cytokine antibody arrays to investigate the role of inflammation in the corneas of KC patients. They discovered the activation of pathways related to wound healing, neuroprotection, angiogenesis, and inflammation. Notably, the authors identified 23 cytokines (including FGF-7, MIP-3α, Flt-3 Ligand, MIP-1δ, IL-3, IL-2, BDNF, IL-4, M-CSF, BMP-4, IL-15, GCP-2, TNF-β, MIF, IL-1ra, Lymphotactin, IL-1α, TGF-β, TNF-α, Angiogenin, MDC, IL-2Rα, I-309) that were significantly elevated, with 15 of these cytokines being exclusively expressed in KC corneas (Loh and Sherwin, 2022). Finally, in 2022, Oltulu et al. demonstrated that the monocyte-to-HDL-cholesterol ratio (MHR) and the neutrophil-to-lymphocyte ratio (NLR) were significantly higher in the blood of KC patients compared to healthy controls, while the lymphocyte-to-monocyte ratio (LMR) was significantly lower. These findings further support the hypothesis of inflammatory pathogenesis for this condition (Oltulu et al., 2022). Moreover, single-cell transcriptomic analysis of cytokine-mediated signaling pathways shows that IL23A and CXCL1 are significantly upregulated in corneal DCs isolated from patients with keratoconus, suggesting that corneal myeloid cells may play a crucial role in this extracellular matrix disorder (Dou et al., 2022).

Known main risk factors include atopy and eye rubbing (Galvis et al., 2017). It has been proposed that any inflammatory comorbidity may add synergistically to other forms of KC-related inflammation and exacerbate its pathogenetic processes (McMonnies, 2015). In allergic conditions, there is a reduction in IL-10, an anti-inflammatory cytokine, along with increased levels of proinflammatory IL-13 and TNF-α (Contreras-Ruiz et al., 2012). These factors may contribute to the pathogenesis of keratoconus; furthermore, studies have shown that post-rubbing tear samples from normal eyes exhibit high concentrations of IL-8, MMP-13, IL-6, TNF-α, and epithelial growth factor compared to contralateral control eyes (Kallinikos and Efron, 2004; Balasubramanian et al., 2013). These elevated levels of inflammatory mediators and growth factors suggest a link between eye rubbing and the progression of keratoconus.

## 5 Oxidative damage and mitochondrial dysfunction link in keratoconus

The cornea is directly exposed to solar UV radiation, which can cause oxidative stress injury due to excess free radicals from air pollution and oxygen, particularly reactive oxygen species (ROS). This could explain why recent studies show that keratoconus samples exhibit higher oxidative stress and lower antioxidant levels than healthy individuals (Navel et al., 2021). However, ROS can be generated from both endogenous and external sources. Exogenous sources of ROS include microbial absorption, nanoparticles, xenobiotics, and radiation. In contrast, endogenous sources of ROS include various cellular organs such as mitochondria, peroxisomes, endoplasmic reticulum, and/or enzymatic processes where oxygen consumption is significant (Table 1) (De Almeida et al., 2022). Although the specific cause of KC has yet to be discovered, there is increasing evidence that oxidative stress and mitochondria are key contributors (Vallabh et al., 2017b). Cumulative oxidative damage and mitochondrial malfunction have been observed in KC cells, including mtDNA deletion accumulation and telomere shortening (Atilano et al., 2005). However, the mechanism that causes mitochondrial malfunction and cell death remains unclear.

**TABLE 1:** Source of cellular oxidative stress and consequences.

Sources of reactive oxygen species	Consequences of reactive oxygen species
NADPH oxidase	Fibrosis
Radiation	Inflammation
Enzymatic and metabolic reactions	Altered intercellular communication
ER stress and unfolded protein response (UPR)	Stem cell exhaustion
Oxidative phosphorylation in mitochondria	Cellular senescence
Cell uptake of microbes, nanoparticles, xenobiotics	Mitochondrial dysfunction
	Telomere shortening
	Genome instability and mutation
	Epigenetic alterations
	Loss of proteostasis
	Disabled macroautophagy
	Deregulated nutrient sensing

Mitochondria are versatile organelles collaborating with host cells to perform biosynthesis, metabolism, and tasks related to cell death or survival. Mitochondria, which are the sites of the tricarboxylic acid (TCA) cycle and oxidative phosphorylation (OXPHOS), produce significant amounts of adenosine 5′-triphosphate (ATP) via the electrochemical gradient of the electron transport chain (ETC). However, mitochondria can also generate ROS, primarily at ETC complexes I and III (Singh et al., 2019). ROS were once considered damaging, but recent research has identified them as emerging key signaling molecules. As a result, in addition to their traditional roles in metabolism, such as glucose oxidation and the synthesis of fatty acids, amino acids, and hormones, mitochondria play active roles in ROS signaling, apoptosis, and innate immunity (Shadel and Horvath, 2015).

Mitochondrial failure can produce unique stress signals. For example, reduced OXPHOS and ETC activity can disrupt mitochondrial ROS (mtROS) generation, abolish mitochondrial membrane potential, or decrease cellular ATP levels, leading to reduced energy production (Napolitano et al., 2021; Perillo et al., 2020). As the primary source of ROS, mitochondria are also prone to becoming ROS targets, which can result in severe effects. Elevated free radicals linked with mtDNA oxidative damage, for example, induce mitochondrial stress and downstream signaling, resulting in cell death (Chen et al., 2018). Finally, the dynamic architecture and distribution of mitochondria inside cells might cause diverse types of stress related to mitochondrial elimination via mitophagy or autophagy (Table 1) (Ma et al., 2020). Mitophagy is a key mitochondrial quality control system that removes undesired or damaged mitochondria and is responsible for basal mitochondrial turnover and the removal of damaged mitochondria during stress (Pickles et al., 2018).

Mitochondria actively engage in the reprogramming of mammalian cells (Quirós et al., 2016; Desai et al., 2020). When DNA is damaged, a significant biological response occurs, including efforts to initiate DNA repair, cell cycle arrest, or death to maintain genomic stability and integrity (Luna-Maldonado et al., 2021). Mitochondrial activity and quality are vital for cellular homeostasis because they create ATP and other necessary components. Mitochondria respond to physiological signals by changing their content, fusion, fission, and unfolded protein response (Leduc-Gaudet et al., 2021). These modifications maintain the energy supply and improve cellular signaling following stress (Leduc-Gaudet et al., 2021). While DNA damage signaling in the nucleus is well understood, less is known about how mitochondria respond to stress, despite their important role in determining cell fate. The number and variety of diseases linked to mitochondrial malfunction emphasize the relevance of mitochondria. During stress, the nucleus and mitochondria interact (Desai et al., 2020). Indeed, in response to endogenous or external perturbations, mitochondria communicate with the nucleus to induce gene transcription. This signaling system, known as the mitochondrial retrograde response (MRR), contributes to the pathophysiology of unregulated cellular proliferation (Strobbe et al., 2021). Lastly, elevated levels of ROS in KC eyes also damage mitochondrial DNA, representing one of the major markers of KC (Atilano et al., 2005).

Damaged mitochondria can induce inflammation. Specifically, the mitochondrial permeability transition pore (mPTP) is a protein complex situated between the inner and outer membranes. When the mPTP opens, it allows leakage of mitochondrial DNA (mtDNA) into the cytoplasm (Yu et al., 2020). BCL-2 Associated X (BAX) and BCL-2 Homologous Antagonist/Killer (BAK) regulate mPTP function during apoptosis by controlling caspase-1 activation leading to the formation of mitochondrial pores (Huang et al., 2020). Moreover, mitochondria-derived vesicles (MDVs) may strategically transport damaged mitochondrial elements to lysosomes for breakdown (Todkar et al., 2021). However, circulating MDVs that contain mtDNA may inadvertently promote inflammation (Picca et al., 2020). While the precise mechanism of mtDNA leakage into the cytoplasm or extracellular space is still unclear the following pathways are suspected:

- cGAS-STING pathway: Cytoplasmic DNA from pathogens is usually recognized as exogenous, prompting an innate immune response. Similarly, mtDNA that leaks from mitochondria and accumulates in the cytoplasm can trigger an inflammatory response via comparable mechanisms (Picca et al., 2020). The type I interferon (IFN) response is vital as a signaling pathway against infections, with STING initially recognized as the protein that facilitates this response. While cytoplasmic DNA activates STING, further research has shown that its ligands encompass cyclic dinucleotides like cyclic dAMP and cyclic dGMP (Picca et al., 2020). STING detects DNA upstream through cyclic GMP-adenosine monophosphate synthase (cGAS). By utilizing ATP and GTP, cGAS binds to cytoplasmic DNA and produces cyclic guanosine monophosphate-adenosine monophosphate (cGAMP), which then activates STING. Following activation, STING moves from the endoplasmic reticulum (ER) to the Golgi apparatus, recruiting TBK1 and IKK. The activated TBK1 and IKK subsequently phosphorylate the downstream targets IRF3 and IκBα, promoting the nuclear translocation of both IRF3 and NF-κB. These transcription factors amplify type I IFN responses and pro-inflammatory cytokine production (Picca et al., 2020).
- Toll-like receptor: Toll-like receptors (TLRs) are conserved pattern recognition receptors crucial for innate immune responses, especially in detecting pathogens in the extracellular matrix. Ten human TLRs (TLR1-TLR10) have been identified, and they are categorized as integrated transmembrane proteins (Bell et al., 2003). The N-terminal domain’s ectodomain recognizes pathogen-associated molecular patterns (PAMPs) and danger-associated molecular patterns (DAMPs), leading to the activation of NF-κB (Vijay, 2018). TLR-9 was the first receptor discovered to sense DNA, predominantly found in the endoplasmic reticulum and later moved to lysosomes upon activation, including hypomethylated CpG motifs (Barbalat et al., 2011). The hypomethylation of mitochondrial DNA allows it to mimic foreign DNA, enhancing its detection by TLR-9 (Hong et al., 2013) promoting inflammatory responses or enhancing type I IFN responses (Schiller et al., 2012).
- Inflammasomes: The innate immune response to PAMPs or DAMPs triggers the activation of inflammasomes, which are composed of receptor, adapter, and caspase-1 proteins (Guo et al., 2015). Mitochondrial dysfunction and electron transport failure cause excessive mtROS, which enhances NLRP-3 inflammasome activation (Zhou et al., 2011). Oxidized mtDNA in the cytoplasm from ATP dysfunction triggers this activation (Shimada et al., 2012). Studies show that mtROS and the NLRP 3 inflammasome promote mtDNA release, increasing IL- 1 β and IL- 18 after LPS or ATP priming (Nakahira et al., 2011). Although mtROS influences inflammasome priming, it is insufficient for full activation, indicating the need for other factors, like mitochondrial membrane potential during viral infections. Calcium signaling triggers NLRP 3 by damaging mitochondria; after ATP activation, Ca^2^ causes harm, leading to mtDNA release and mtROS production. Various activators mobilize Ca^2^ and cause mitochondrial damage via calcium overload (Ichinohe et al., 2013).

Thus, mitochondrial dysfunction in KC may be associated with inflammation through various pathways triggered by oxidative stress.

## 6 Senescence as key drive of keratoconus

KC corneas exhibit higher amounts of mtDNA damage than normal corneas (Atilano et al., 2005). The previously established alterations in mtDNA integrity and increased oxidative stress may be linked and contribute to KC pathogenesis and inflammation. DNA damage caused by a redox imbalance in the nucleus or mitochondria activates the DNA damage response (DDR) (Rinaldi et al., 2024). ATM and ATR kinases control this DDR, which affects gene expression and metabolism, culminating in a senescent phenotype (Maréchal and Zou, 2013). The signaling proteins p53, p16, and p21 induce DDR-mediated senescence by halting the cell cycle in G1 or G2 and enhancing cytokine release, resulting in the senescence-associated secretory phenotype (SASP) (Roger et al., 2021; Pezone et al., 2023). SASP is characterized by mitochondrial dysfunction and cytokine secretion, which indicate DNA damages (Khavinson et al., 2022). Numerous biomarkers, such as p16, p21, and SA-βgal (Gu et al., 2020), indicate cellular senescence. Immunological and parenchymal cell senescence not only accelerates aging but also plays a role in the emergence of numerous illnesses and metabolic conditions (Childs et al., 2015).

The broad phenomena of cellular senescence influence tissue remodeling, including embryogenesis and wound healing. Cell cycle arrest is brought about by the production of inflammatory cytokines with paracrine, autocrine, and endocrine effects during cellular senescence. Morphological changes in senescent cells include flattened cell bodies, aberrant organelles, cytoplasmic vacuolization and granularity, and ECM remodeling (Boccardi et al., 2024). The ECM is a dynamic structural network that maintains normal tissue homeostasis via biochemical and physical scaffolding. ECM disruption has been linked to a variety of clinical conditions. Senescent cells, on the other hand, have a distinct secretory phenotype that modifies their microenvironment and changes the composition and organization of the ECM (Mavrogonatou et al., 2023). Senescence-related changes in the extracellular matrix (ECM) are mainly marked by altered expression levels of fibronectin, collagen I, and collagen III (Mavrogonatou et al., 2019). Additionally, fibroblasts that are senescent from diverse sources tend to overexpress various matrix metalloproteinases (MMPs) including MMP-1, -3, -8, -10, -11, -12, and -13, as well as cathepsin O, urokinase-type plasminogen activator (uPA), tissue plasminogen activator (tPA), and plasminogen activator inhibitor (PAI)-1 and -2, while showing a downregulation of their tissue inhibitors (TIMPs) (Mavrogonatou et al., 2019). This results in changes to both the composition and organization of the ECM.

A variety of conditions that also contribute to mitochondrial malfunction cause cellular senescence. A protective impact against senescence and the development of pro-inflammatory SASP markers is associated with reductions in ROS and nuclear DNA damage foci, particularly telomere-associated foci, in a transgenic cell culture model of mitochondrial elimination by mitophagy (Wiley and Campisi, 2021). Parallel to this, mitochondrial ROS can activate the family of protein kinases known as c-Jun N-terminal kinases (JNKs), which are essential in stress signaling pathways. This activation increases proinflammatory SASP component activation and cytosolic chromatin fragment release (Miller et al., 2021). Based on this and other studies, mitochondrial ROS may significantly influence cellular senescence. Indeed, mitochondrial dysfunction-associated senescence (MiDAS) occurs when mitochondrial disfunction disrupts cellular metabolism and increases reactive oxygen species (ROS) (Wiley et al., 2016). Oxidative phosphorylation (OXPHOS) in the mitochondria utilizes the electron transport chain (ETC) located in the inner mitochondrial membrane to produce energy. Due to mitochondrial dysfunction and issues with the ETC, electron leakage at Complexes I and III causes mitochondrial dysfunction and ETC dysfunction (Zong et al., 2024). Electron leakage partially reduces oxygen, generating ROS. At low levels of ROS, they serve as signaling molecules, and excessive production can damage DNA, proteins, and lipids. This oxidative damage activates the DNA damage response (DDR) pathway, stabilizing tumor suppressor proteins such as p53 and initiating cell cycle inhibitors like p21, leading to persistent cell cycle arrest and senescence (Shreeya et al., 2023). With mitochondrial failure, senescent cells switch to glycolysis (the Warburg effect), which hinders OXPHOS efficiency and alters mitochondrial biogenesis. Continuous production of ROS amplifies the senescence-associated secretory phenotype (SASP), resulting in inflammation, tissue dysfunction, and aging. The interplay between mitochondrial dysfunction and ROS overproduction drives senescence, linking mitochondrial health to cellular aging and age-related diseases. Furthermore, senescent cells can accumulate and release the SASP, a complex mixture of pro-inflammatory cytokines, growth factors, and proteases. This secretory profile can modify the tissue microenvironment, influence angiogenesis, and facilitate epithelial-mesenchymal transition (EMT) (Qin et al., 2025).

Additionally, MiDAS is characterized by a low NAD+:NADH ratio in its metabolic profile. NAD+ serves as a cofactor for poly (ADP-ribose) polymerases (PARPs), which are involved in single-strand break (SSB) repair, and sirtuins that help maintain mitochondrial integrity. A deficiency in NAD+ hampers DNA repair and leads to SSB accumulation, activating ATR and p53 pathways and causing growth arrest (Wiley et al., 2016; Xie et al., 2020).

(Wiley et al., 2016) Mutations that disrupt the proofreading domain of mitochondrial DNA polymerase, such as PolgD257A, can cause mitochondria-induced senescence, resulting in progeroid mice with mitochondrial DNA mutations and aging characteristics in most tissues. As mentioned earlier, because mitochondria oxidize NADH to NAD+ and because mitochondrial failure lowers the NAD+:NADH ratio, the metabolite NAD+ is the main node in mitochondria-induced senescence (Vasileiou et al., 2019; Trinchese et al., 2024). In addition to oxidizing NADH from the fatty acid oxidation or tricarboxylic acid cycle, mitochondria additionally use the malate-aspartate and α-glycerophosphate shuttles to oxidize the cytosolic NAD+-NADH pool. Depletion of malate dehydrogenase inhibits the latter, lowering the NAD+:NADH ratio and inducing senescence, implying that increased NAD+ levels prevent senescence (Chini et al., 2024).

One sign of metabolic disturbance during cell senescence is the loss of molecular and protein homeostasis. Numerous processes, such as the DDR brought on by telomere attrition, decreased tricarboxylic acid cycle activity, mitochondrial dysfunction leading to ATP production, reduced degradation of the proteasome and autophagolysosome, alterations in SASP, and epigenetic modification, are responsible for the remodeling of metabolic signals and metabolites in cells (Zhu et al., 2021; Russo et al., 2021; Improda et al., 2023; Pezone et al., 2017; Nappi et al., 2023).

A distinction between the central and peripheral corneal cells is highlighted by the fact that the central cornea in KC is thinner than the peripheral cornea and is more prone to scarring (Li et al., 2023; Brautaset et al., 2013). Previous findings indicated that the KC peripheral cells had greater amounts of the senescence-related genes p21, p27, and p53. The tumor suppressor protein p53, which is involved in cell death, senescence, genomic stability, and suppression of angiogenesis, tightly regulates the expression of p21 and p27 (Li et al., 2023). p21 stops growth and keeps the cell cycle in the G1/S phase. Peripheral keratocyte senescence may be partially attributed to the increased expression of these genes.

KC patients with elevated levels of inflammatory cytokines also displayed symptoms of oxidative stress, mitochondrial dysfunction, and several differentially methylated areas (Mavrogonatou et al., 2023; Kabza et al., 2019).

**FIGURE 3:**
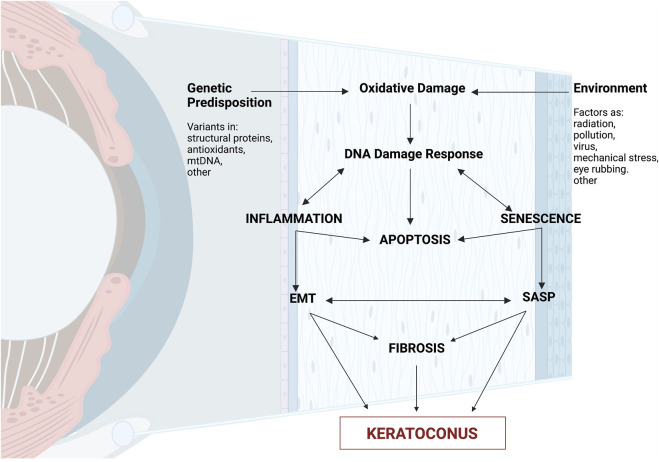
The cascade of molecular processes in the pathophysiology of keratoconus. Summary of molecular involvement in the corneal layers. External causes such as eye rubbing, increased reactive oxygen species (ROS), mitochondrial damage, or gene alterations can activate biochemical cascades, causing a lack of cellular homogeneity in the corneal epithelium. There is an increase in proinflammatory cytokines (IL-6, TNF-alpha) and an exacerbation of the function of metalloproteinases (MMP-1, MMP-2, MMP-9, MMP-13), resulting in increased keratocyte apoptosis, resulting in a reduction in corneal stromal thickness, and both histological changes and disruption of the extracellular matrix. Created with Biorender.com.

As a result, cellular senescence, a process that imposes a permanent proliferative arrest on cells in response to various stressors, induces premature aging in KC (Childs et al., 2015). Thus, we propose a model in which the end products of the DNA damage response are growth factors, proteases (SASP), immune modulators, and inflammatory cytokines. This leads to progressive reorganization of the cytoskeleton network (actin and microtubules) and fibrosis, causing corneal thinning and ECM degradation–hallmark features of KC – even though the pathogenic mechanism and source of DNA damage in the KC corneal epithelium are unknown (Figure 3).

## 7 Conclusion and future perspectives

High levels of oxidative stress are characteristic of KC; regardless of the source of the oxidative damage, KC can result in significant and/or chronic DNA damage that can cause senescence and SASP. Moreover, patients’ inflammatory markers were discovered (Forman and Zhang, 2021). In this study, we emphasize how keratoconus pathogenesis exhibits senescence (Figure 3). Considering that corneal cells are highly susceptible to changes in cytoskeletal stability and DNA integrity, it may be concluded that oxidative damage causes inflammation and ultimately leads to their mortality. This provides new insight into the molecular pathophysiology of KC and opens up new avenues for treatment and monitoring of the condition. The senescence response may be advantageous or detrimental depending on the physiological context (Wiley and Campisi, 2021). Senescent cells are temporarily found in tissue injury areas and play a role in wound healing, tissue repair, and regeneration. This is probably due to certain SASP factors (Pezone et al., 2023; Chu et al., 2020).

Early keratoconus treatment starts with spectacles for vision improvement, progressing to rigid gas-permeable contact lenses as the disease advances. Some patients may eventually need corneal transplantation. New therapies like refractive, optical, and lamellar surgery can slow disease progression and delay intensive treatments. Collagen crosslinking (CXL) with ultraviolet A (UV-A) light and riboflavin (vitamin B2) is a newer method shown to slow early-stage disease progression.

Advanced collagen cross-linking strategies: a new avenue for halting keratoconus progression is based on the idea of leveraging the cornea’s self-repair abilities. The IVMED-80 method utilizes the cornea’s self-repair capabilities to slow KC progression. Intrastromal corneal ring segments (ICRS) reduce distortion, relying on proper positioning and diameter. Corneal allogenic intrastromal ring segments (CAIRS) use allogeneic tissue for outcomes similar to synthetic ICRS, with a doughnut variant for moderate to advanced keratoconus. Topography-guided custom ablation employs corneal topography for laser ablation, improving visual acuity in advanced cases pre- and post-surgery. Corneal transplantation replaces a damaged cornea with a healthy donor one, with penetrating keratoplasty (PK) as the conventional full-thickness transplant for advanced cases, while deep anterior lamellar keratoplasty (DALK) yields better outcomes. Bowman layer transplantation (BLT) is an option when CXL or ICRS aren’t feasible, achieving corneal flattening and 84% progression-free survival over 5 years.

Future directions may explore novel treatments for keratoconus, focusing on reducing ROS levels. Antioxidants could play a vital role in countering free radicals and protecting cellular health, although traditional antioxidants have demonstrated limited benefits in clinical trials. Other promising strategies are being investigated in various diseases.

- Mitochondrial-antioxidants: Mitoquinone (MitoQ) is a non-targeted antioxidant effective against kidney diseases, metabolic syndromes, systemic inflammatory response syndrome, cardiovascular issues, eye disorders, arthritis, and aging. Its safe oral administration makes MitoQ a dietary supplement. It neutralizes reactive oxygen species (ROS) like superoxide, peroxyl, and peroxynitrite in mitochondria (Fields et al., 2023). In contrast, SkQ1 is a mitochondria-targeted antioxidant with similar activity, featuring the mitochondria-targeting molecule TPP linked to plastoquinone. SkQ1 is used in cosmetics for anti-aging, prevents UV-induced corneal damage, and promotes wound healing after ocular surgery. It reduces oxidative stress in corneal epithelial cells and aids endothelial cells in healing. This indicates SkQ1’s potential as premedication during ocular surgery to prevent iatrogenic corneal complications (Zernii et al., 2018).
- NAD+ restoration strategies: Cellular senescence, mitochondrial dysfunction, and nutrient sensing are pivotal research areas, with low NAD+ (nicotinamide adenine dinucleotide) identified as a key contributor to aging signs. Interventions aim to prevent or reverse aging changes while stimulating pathways for healthier living (Guo et al., 2022). NAD+ is central for ATP production and as a cofactor for enzymes, especially sirtuins. SIRT1 deacetylates protein substrates, releasing nicotinamide (NAM) (Covarrubias et al., 2021). Other crucial enzymes like PARPs promote lifespan, while CD38, a primary NAD+ hydrolase, participates in various cellular functions. The interest in enhancing NAD+ as an anti-aging approach has increased, given its instability and low bioavailability. Supplements offer precursors for the salvage pathway, mainly NR or NMN (Chini et al., 2024). A major challenge is the age-related decline in NAD+-metabolizing machinery, affecting production and use. Thus, KC relates to lower NAD+ levels that worsen senescence, emphasizing the need for NAD+ restoration through small-molecule inhibitors, activators, or dietary supplements like NMN and NR’ (Xie et al., 2020).
- Anti-Ageing Therapy: Senolytics are drugs studied for treating degenerative diseases by preventing cellular senescence, which causes fibrosis, neurological issues, and chronic diseases. Therapies include senolytic drugs targeting senescent cells and senomorphic drugs acting indirectly. They help mitigate aging effects, reduce inflammation, and influence tumor development. Notable examples include natural compounds, kinase inhibitors, and various mimetics. Senotherapeutics, like rapamycin and metformin, lower SASP secretion without removing senescent cells. Some compounds, such as procyanidin C1, extend mice lifespan by targeting senescence and inducing apoptosis at higher doses. Natural compounds, including Apigenin, EGCG, and quercetin, regulate senescence through different pathways. Senotherapeutics show promise for breast cancer by targeting SASP. One inhibitor blocks mTOR, reducing growth and reversing senescence-associated phenotypes. Rapamycin shifts senescence to a quiescent state in MCF-7 cells. Another small-molecule inhibitor selectively eliminates senescent cells and reduces SASP factors. Metformin prevents senescence by blocking NF-κB nuclear translocation (Lelarge et al., 2024; Chaib et al., 2022).

## 8 Methods of search

For this review, we extensively explored the literature by utilizing the PubMed, Scopus, and Cochrane databases. Our search methodology comprised a blend of keywords aimed at encompassing pertinent studies, such as “keratoconus,” “inflammation,” “oxidative stress,” “DNA damage,” “senescence,” “mitochondrial dysfunction,” and combinations of them. The articles identified underwent meticulous review and analysis, synthesizing a thorough understanding of what’s known in this domain of study.
